# Environmental Factors Associated with Type 1 Diabetes Development: A Case Control Study in Egypt

**DOI:** 10.3390/ijerph14060615

**Published:** 2017-06-07

**Authors:** Nabil J. Awadalla, Amal A. Hegazy, Manal Abd El-Salam, Marwa Elhady

**Affiliations:** 1 Department of Family and Community Medicine, College of Medicine, King Khalid University, Abha 61421, Saudi Arabia; 2 Department of Community Medicine, Faculty of Medicine, Mansoura University, Mansoura 3551, Egypt; 3 Department of Family and Community Medicine, Faculty of Medicine, King Abdulaziz University, Jeddah 21589, Saudi Arabia; renalahmed@gmail.com; 4 Department of Community and Occupational Medicine, Faculty of Medicine (for girls), Al-Azhar University, Cairo 11651, Egypt; 5 Department of Pediatrics, Faculty of Medicine (for girls), Al-Azhar University, Cairo 11651, Egypt; manal24969@gmail.com (M.A.E.-S.); marwaelhady93@yahoo.com (M.E.)

**Keywords:** type 1 diabetes, environment, risk factors, Egypt, children

## Abstract

Uncertainty still exists regarding the role of some environmental risk in the development of type 1 diabetes mellitus (T1DM) both globally and in Egypt. The objective here was to explore the potential environmental risk factors associated with the development of T1DM among children in Egypt. A case-controlled study of 204 T1DM children and an equal number of age and sex-matched controls was conducted in Assiut, Egypt. Data regarding the parental, gestational, neonatal, and childhood possible risk factors for T1DM were evaluated. The final sex adjusted multivariable logistic regression model revealed that the risk for T1DM was significantly higher among rural residents (aOR = 2.03, 95% CI: 1.30–4.25), those with parental history of T1DM (aOR = 9.03, 95% CI: 1.02–83.32), birth through cesarean section (aOR = 2.13, 95% CI: 1.09–5.03), and having history of early introduction of cow milk in the first year of life (aOR = 19.49, 95% CI: 8.73–45.53). On the other hand, a protective effect was observed between at least six months’ breastfeeding, vitamin D supplementation in the first year of life, high physical activity, and the development of T1DM. Educational programs should be adopted to improve awareness and knowledge of the parents to avoid the increased risk factors and encourage protective practices.

## 1. Introduction

Type 1 diabetes mellitus (T1DM) is an autoimmune disease that cause selective loss of pancreatic beta cells [1]. It is one of the most important chronic diseases of children. It affects more than 35 million people worldwide, and its incidence shows significant geographic variability, ranging from less than 1/100,000 to more than 40/100,000 per year [2]. In the Middle East and North Africa, about 129,000 children with T1DM, the largest contributions to this number come from Saudi Arabia and Egypt whose estimates jointly account for nearly half of the region’s total. The incidence of T1DM in Saudi Arabia (31.4 per 100,000 population), Kuwait (22.3 per 100,000 population), and Egypt (8.0 per 100,000 population) [2].

Although the genetic role plays an important role in the development of T1DM, the rapid changes in incidence rate reported to occur within comparatively short time periods are more likely owing to changes in environmental risk factors [2,3]. These environmental risk factors may act through initiation of autoimmunity or acceleration and precipitation of an already ongoing beta cell damage [4]. These potential risk factors include; early life consumption of cows’ milk [5], viral infections during pregnancy and postnatally [6], older maternal age [7], and caesarean section delivery [8]. On the other hand, systematic reviews of observational studies have recognized some protective factors including; breastfeeding [9], early life vitamin D supplementation [10], and early childhood infections [11].

Studies addressing the link between environmental factors and T1DM among children in Egypt are so far scarce. Knowledge of the T1DM modifiable environmental risk factors in Egyptian children can help the authorities in planning and implementing preventive polices to reduce the burden of disease. Therefore, the aim of this study was to explore the potential environmental risk factors associated with the development of T1DM among children in Egypt.

## 2. Subjects and Methods

A case-control study was carried out in pediatric clinics at the Health Insurance Hospital, Assiut City, Egypt, over the period of four months from March to July 2016. This hospital is the largest public hospital in Assiut City and serves a large population, contains multiple specialties in medicine, and has 650 beds. Parents of all studied children gave informed written consent before the involvement in the study. The study was conducted in accordance with the Declaration of Helsinki, and the protocol was approved by the Ethics and Research Committee of the College of Medicine of Al-Azhar University. The ethics code of the research is No. 1432, date 11 December 2015). Written approval was obtained from the administration of Assiut Health Insurance Hospital before starting the study.

Using the WHO manual for sample size determination in health studies, the sample size determined by using the proportion difference approach with the assumption of 95% confidence level, 80% power, control to case ratio 1:1, the odds ratio to be detected ≥2 and the 20% control group will be exposed [12]. The minimal sample size required 171 cases and 171 controls with total of 342. To account for possible non-response and for more precision, 20% was added to give a total sample of 408 children (204 cases and 204 controls).

The case group comprised 204 children in the age group of 6–16 years who were diagnosed with T1DM based on WHO criteria [13] and who were receiving care at Assiut Health Insurance hospital. The cases were randomly selected form the children attending diabetes pediatric clinics through systematic random sampling technique. The diabetes clinic is held twice a week and the average number of diabetic patients attending per day is 15 patients. Every other patient was selected to get the required sample size. The control group involved equal number of non-diabetic children chosen from other pediatric clinics of the same hospital by selecting for every case a matched age and sex control. T1DM was excluded from controls by history taking and measuring their random blood sugar. The response rates were 95% and 87% among cases and controls respectively.

### 2.1. Study Tools

A structured questionnaire was constructed using Delphi technique by the researchers after reviewing the literature related to environmental risk factors and T1DM [3,4,5,6,7,8,9]. The questionnaire was developed and validated by experts in epidemiology and pediatrics. It was designed for the purpose of the study and data were collected by interviewing both the child and parents. It includes the following information: (a) demographic data such as, name, age, sex, residence, age at diagnosis of T1DM and family income; (b) parental characteristics, such as age of mother at delivery of the child, education, occupation, smoking, consanguinity, and history of diabetes for both of them; (c) maternal factors during pregnancy which include preeclampsia, gestational diabetes, autoimmune diseases, amniocentesis, maternal viral infections, maternal bacterial diseases, antepartum hemorrhage, abnormal weight gain during pregnancy, previous abortions, previous fetal death, mode of delivery, and drug intake during pregnancy; (d) natal and early neonatal characteristics such as mode and place of delivery, pregnancy duration, weight at birth (kg), birth order, history of neonatal jaundice, neonatal infection, and neonatal respiratory distress; (e) feeding pattern in first year of life as breast feeding, length of exclusive breast feeding, overall duration of breast feeding, introduction of cows’ milk in first year of life, onset of weaning, onset of meat, fish, fruit, and eggs introduction and vitamin D supplement; (f) early childhood environmental exposures such as history of infectious diseases, autoimmune diseases, thyroid disease, rheumatic disease, rhinitis, eczema and asthma, the consumption pattern of different food staffs, stressful events, and level of physical activity.

### 2.2. Data Entry and Analysis

The obtained data were entered, revised, and analyzed using SPSS, version 22 software package (IBM, North Castle, NY, USA). Binary sex adjusted multivariable logistic analysis, adjusted odds ratios (aORs), and antecedent 95% confidence intervals (95% CIs) were used to identify the potential risk factors of T1DM. The dependent variable was having diagnosed T1DM. The independent variables included in the present study were entered in four models; maternal and environmental factors occurring during pregnancy model, natal and neonatal environmental exposures model, feeding practices in the first year of life model, and childhood environmental factors model. Then, the significant factors associated with T1DM in each model entered in the final backward stepwise model. Logistic regression explores the relationship between the dependent variables and the independent variables by measuring probabilities using a logistic function.

## 3. Results

### 3.1. Description of the Study Sample

The current study comprised 408 children. These included 204 Type 1 DM cases; 100 (49.0%) boys and 104 (51.0%) girls with average age of 11.3 years (SD 3.8 years). The mean age at the onset of diagnosis was 7.94 years (3.39 years) and the mean duration of diabetes was 6.36 years (4.46 years). The controls were selected by individual age and sex matching (Table 1).

### 3.2. Maternal and Environmental Factors Occurring during Pregnancy

The sex adjusted multivariable logistic regression model for the maternal and environmental factors occurring during pregnancy (Table 2) showed that being resident in rural area (OR = 1.56, 95% CI: 1.02–2.50) was a significant risk factor for having T1DM. Similarly, the presence of the subsequent risk factors were detected significant for having T1DM: insufficient family income, mother’s age above 35 years, family history of T1DM, consanguinity, and maternal infection and overweight gain during pregnancy. However, the only significant risk factors observed with multivariable logistic regression model for the significant factors in maternal, natal, neonatal, first year feeding practices and childhood environmental factors models were being a rural resident (aOR = 2.03, 95% CI: 1.30–4.25) and having parental history of T1DM (aOR = 9.03, 95% CI: 1.09–85.32) (Table 6).

### 3.3. Natal and Neonatal Factors

The sex adjusted multivariable logistic regression model for the natal and neonatal environmental factors (Table 3) revealed that hospital delivery vs. home delivery (aOR = 0.15, 95% CI: 0.08–0.31) minimizes the risk of T1DM. On the other hand, positive history of neonatal jaundice (aOR = 5.62, 95% CI: 2.98–10.62) was a significant risk factor for having T1DM vs. negative. Similarly, positive history of preterm birth, birth through cesarean section, and second and greater birth order were associated with higher risk of T1DM. Yet, the only significant risk factor detected with multivariable logistic regression model for the significant factors the maternal, natal, neonatal, first year feeding practices and childhood environmental factors models was birth through cesarean section (aOR = 2.13, 95% CI: 1.09–5.03) Table 6.

### 3.4. Feeding Practices in the First Year of Life

The sex adjusted multivariable logistic regression for the feeding practices in the first year of life (Table 4) showed that positive history of breast feeding more six months (aOR = 0.10, 95% CI: 0.07–0.48), and vitamin D supplementation during the first year of life (aOR = 0.14, 95% CI: 0.08–0.24) were significant protective factors against T1DM. On the other hand, a positive history of introduction of cow milk in the first year of life (aOR = 6.37, 95% CI: 3.23–12.58) was a significant risk factor. The final multivariable logistic regression model for the significant factors in maternal, natal, neonatal, first year feeding practices, and childhood environmental factors models confirms the protective effect of more than six months’ breast feeding and vitamin D supplementation during the first year of life as well as the risky effect of introduction of cow milk in the first year of life (Table 6).

### 3.5. Childhood Environmental Factors

The sex adjusted multivariable logistic regression model for the childhood environmental factors (Table 5) revealed that positive history of subjective perception of high frequent intake of sweet (aOR = 3.35, 95% CI: 1.95–5.76) was significant risk factor of T1DM. While history of frequent intake of vegetables (>4 times/week) (aOR = 0.24, 95% CI: 0.09–0.70) and subjective perception of high physical activity (aOR = 0.18, 95% CI: 0.13–0.25) were significant protective factors. The final multivariable logistic regression confirms only the protective effect of high physical activity (Table 6).

## 4. Discussion

Several studies have incriminated many environmental factors in the development of T1DM [3,4,6,7,8,9]. The controversy that exists between the results of these studies makes it difficult to obtain a definite conclusion in this regard. Furthermore, the global distribution of childhood T1DM shows great area-to-area variations. This variability between countries and regions may be explained partly by different distributions of T1DM risk genes beside different distributions of environmental exposures [2]. The present study provides some evidence regarding the environmental risk factors of T1DM among children in Assiut City, the largest city in Upper Egypt.

The final multivariable logistic regression model for the significant factors in maternal, natal, neonatal, first year feeding practices and childhood environmental factors models revealed that the risk T1DM was significantly higher among rural residents, those with parental history of T1DM, birth through cesarean section, and having history of early introduction of cow milk in the first year of life. On the other hand, a protective effect was observed between six months’ breastfeeding, vitamin D supplementation in the first year of life, high physical activity, and the development of T1DM.

The present study founds a positive association between rural residence and higher risk of T1DM in the final multivariable analysis model. This finding is in accordance with studies conducted in Finland [14] and USA [15] but was inconsistent with the finding from a global study [2], India [16], and Italy [17] where children living in the rural areas had a lower risk for T1DM. The higher risk of type 1 diabetes among rural residents observed in the current study can be difficultly explained by urban/rural difference in distribution of risky genes but by environmental exposures [14]. Rapid urbanization observed in the Egyptian rural communities in recent decades may be considered as an indirect factor for developing unhealthy, deprived, high density informal areas within the rural communities and neighborhoods of large cities [18]. These areas suffered from many environmental problems such as overcrowding, air pollution from increased number of vehicles, industrialization and lack of green areas, lack of proper infrastructure, and inadequate social and health services [18]. These environmental factors—besides lower mother’s education, earlier cow milk introduction, lower level of health care, and repeated infections—may explain the unexpected higher risk of T1DM in the studied Egyptian rural communities [6,11,15].

Our result confirms previous studies in Germany [7], UK [19], and Serbia [20] which report that T1DM is significantly associated with positive family history of T1DM. This positive association confirms the role of inheritance in the pathogenesis of TDM [21].

Birth through cesarean section was observed to be a significant predictor positively associated with T1DM in the present study by the final multivariable analysis. This finding is in accordance with that stated by a systematic review conducted by Patelarou et al. [22].

History of neonatal jaundice was found as a significant risk factor by multivariable analysis of natal and neonatal factors and insignificantly associated with a higher risk of T1DM by multivariable logistic analysis for the significant factors in all models. This finding is consistent with the result of Scotch matched case control study [23] as well as a meta-analysis study conducted by McNamee et al. [24] which reported insignificant higher risk of T1DM with history of neonatal jaundice.

The final multivariable analysis model in the current study revealed a protective association between breastfeeding for at least six months and development of T1DM this is in agreement with the protective association reported by a systematic review conducted by Patelarou et al. [22]. Most of the studies in this systematic review considered that absence of breastfeeding or breastfeeding for a short period a major risk factor for the development of T1DM. The protective effect of breast milk may be attributed to its content of efficient antimicrobial substances which protect against a wide range of microorganisms [22]. Consequently, breast feeding seems to guard infants from infections with enteroviruses and accordingly from B cell autoimmunity that progressively could result in the development of T1DM [25]. On the other hand, the present study provides adequate evidence that early introduction of cow milk in the first year of life was associated with a higher risk of T1DM. This finding is in agreement with that conducted in Finland [26] and Iran [3] and a review article conducted by Vertanen and Knip [27]. The diabetogenic effect of cow milk could be explained by early immunization to bovine insulin that occurs in some persons. Whether this initiates autoimmune response to human insulin and insulin producing B cells remains to be confirmed [28,29].

Vitamin D supplementation in the first year of life was found to be a significant protective factor against T1DM in the present study by both initial and final multivariable analysis. The preventive role of vitamin D supplementation has been reported by meta-analysis study conducted by Zipitis and Akobeng [10]. The protective effect of vitamin D could be explained by the downregulation of aggressive autoimmune response [30].

The current study found a significant protective effect of participating high level of physical activity and lower risk of T1DM. This could be related to enhanced insulin sensitivity and decreased B-cell workload [31].

The present study provides new insight about the environmental factors associated with the development of T1DM among children in Assiut, Upper Egypt. The experiences obtained could be generalized in similar communities to minimize the risk of T1DM. Yet, it has some limitations related to the case control study design. These include liability for recall bias, selection bias, and confounding. To compensate for selection bias, an age and sex matched control child was selected for every case from the same study pool. To account for the confounding, multivariable logistic regression was done for each domain separately and a final model for the significant factors.

## 5. Conclusions

The current study recognizes the significant environmental factors associated with the development of T1DM in Assiut, Egypt: these include being a resident of rural areas, having parental history of T1DM, birth through cesarean section, and early exposure to cow milk in the first year of life. Moreover, the study demonstrates the protective role of breastfeeding, vitamin D supplementation in the first year of life, and physical exercise. Well-designed cohort and experimental studies are required to confirm these results. Educational programs should be adopted to improve awareness and knowledge among parents about the importance of practicing protective measures as breast feeding, vitamin D supplementation during the first year of life, and physical exercise and, when possible, avoiding the exposure to risk factors such as caesarian delivery and early introduction of cow milk. Also, measures should be taken for more development of rural areas though proper planning, combating air pollution, and improving infrastructure and health services.

## Figures and Tables

**Table 1:** Characteristics of the study population.

Variables	Cases *n* = 204	Controls *n* = 204
Sex		
Male	100 (49.0%)	100 (49.0%)
Female	104 (51.0%)	104 (51.0%)
Age (years)		
6–9	116 (56.86%)	116 (56.86%)
10–16	88 (43.14%)	88 (43.14%)
Age at diagnosis of T1DM (years)	7.94 ± 3.39	-
Duration of T1DM (years)	6.36 ± 4.46	-

**Table 2:** Sex adjusted multivariable logistic regression for the maternal factors predicting T1DM.

Maternal Factors	aOR (95% CI)
Residence (rural vs. urban)	**1.56 (1.02–2.50)**
Family income (insufficient vs. sufficient)	**2.37 (1.41–3.99)**
Mother’s age (>35 years vs. <35 years)	**8.53 (1.87–38.97)**
Family member smoking (positive history vs. negative)	1.01 (0.85–1.89)
Family history of type 1 DM (positive history vs. negative)	**2.48 (1.23–34.72)**
Consanguinity (positive history vs. negative)	**2.01 (1.32–4.78)**
History of maternal diseases/exposures during pregnancy	
Pre-eclampsia (positive vs. negative)	1.83 (0.26–7.83)
Gestational DM (positive vs. negative)	0.98 (0.38–2.52)
Maternal infection (positive vs. negative)	**2.89 (1.02–10.76)**
Antepartum hemorrhage (positive vs. negative)	1.21 (0.43–3.40)
Over weight gain during pregnancy (positive vs. negative)	**2.94 (1.10–8.95)**
Antibiotic intake during pregnancy (positive vs. negative)	2.45 (0.59–10.98)
Antihypertensive intake during pregnancy (positive vs. negative)	1.50 (0.26–8.62)
Antiemetic intake during pregnancy (positive vs. negative)	0.93 (0.43–2.15)

aOR = Sex adjusted Odds Ratio for studied maternal factors (95% CI) = 95% Confidence Interval. Bold aOR (95% CI) are statistically significant.

**Table 3:** Sex adjusted multivariable logistic regression for the natal and neonatal factors predicting T1DM among the study population.

Natal and Neonatal Factors	aOR (95% CI)
Place of delivery (hospital vs. home)	**0.15 (0.08–0.31)**
Mod of delivery (cesarean vs. vaginal)	**2.81 (1.59–4.95)**
Duration of pregnancy (preterm vs. full-term)	**4.60 (1.21–17.49)**
Birth weight (Kg)	
Birth weight (2.5–3.0 vs. <2.5)	0.77 (0.30–2.01)
Birth weight (>3 vs. <2.5)	0.94 (0.34–2.62)
Birth order	
Birth order (second vs. first)	**2.16 (1.21–3.85)**
Birth order (third or more vs. first)	**2.00 (1.05–3.80)**
History of neonatal jaundice (positive vs. negative)	**5.62 (2.98–10.62)**
History of neonatal infection (positive vs. negative)	0.34 (0.07–1.68)
History of neonatal respiratory distress (positive vs. negative)	0.43 (0.09–1.56)

aOR = Sex adjusted Odds Ratio for studied natal and neonatal factors (95% CI) = 95% Confidence Interval. Bold aOR (95% CI) are statistically significant.

**Table 4:** Sex adjusted multivariable logistic regression for the feeding practices in the first year of life predicting T1DM feeding practice.

Feeding Practice	aOR (95% CI)
Breast feeding (>6 months vs. <6 months)	**0.10 (0.07–0.48)**
History of introduction of cow’s milk in first year of life (positive vs. negative)	**6.37 (3.23–12.58)**
History of vitamin D Supplementation in first year of life (positive vs. negative)	**0.14 (0.08–0.24)**
Onset of weaning (less than five months vs. more than five months)	1.25 (0.24–1.71)

aOR = sex adjusted Odds Ratio for studied feeding practices (95% CI) = 95% Confidence Interval. Bold aOR (95% CI) are statistically significant.

**Table 5:** Sex adjusted multivariable logistic regression for the childhood environmental factors predicting T1DM.

Childhood Environmental Factors	aOR (95% CI)
**Childhood infection (positive history vs. negative)**	
Rubella	1.02 (0.01–86.72)
Measles	7.89 (0.49–127.14)
Varicella	4.62 (0.59–13.42)
Mumps	3.65 (0.56–20.53)
**Allergy (positive history vs. negative)**	
Eczema	1.88 (0.61–5.74)
Rhinitis and conjunctivitis	1.39 (0.45–4.29)
Bronchial asthma	1.33 (0.38–4.71)
**Diet (positive history vs. negative)**	
Frequent intake of preserved meat (>4 times/week)	0.59 (0.36–1.95)
Frequent intake of sweets (high)	**3.35 (1.95–5.76)**
Frequent intake of meat (>4 times/week)	1.25 (0.52–3.01)
Frequent Intake of fish (>4 times/week)	3.66 (0.77–17.35)
Frequent Intake of vegetables (>4 times/week)	**0.24 (0.09–0.70)**
Moving home (positive history vs. negative)	1.75 (0.11–27.10)
Physical activity (high vs. low)	**0.18 (0.13–0.25)**

aOR = Sex adjusted Odds Ratio for studied childhood environmental factors (95% CI) = 95% Confidence Interval. Bold aOR (95% CI) are statistically significant.

**Table 6:** Multivariable logistic regression for the significant maternal, natal, neonatal and childhood environmental factors predicting type1 DM.

Factors	β	S.E.	aOR (95% CI)
Residence (rural vs. urban)	0.79	0.36	**2.03 (1.30–4.25)**
Parental history of T1DM (positive history vs. negative)	3.90	2.54	**9.03 (1.02–85.32)**
Mod Of Delivery (cesarean vs. vaginal)	0.75	0.43	**2.13 (1.09–5.03)**
Breast feeding (>6 months vs. <6 months)	−1.46	0.39	**0.23 (0.11–0.50)**
Introduction of cows milk in first year of life (positive vs. negative)	2.99	0.42	**19.94 (8.73–45.53)**
Vitamin D Supplementation in first year of life (positive vs. negative)	−2.16	0.37	**0.11 (0.05–0.24)**
Physical Activity (high vs. low)	−2.90	0.44	**0.05 (0.02–0.12)**

β = Beta coefficients, aOR = Sex adjusted Odds Ratio for studied childhood environmental factors (95% CI) = 95% Confidence Interval. Bold aOR (95% CI) are statistically significant. Family income, mother’s age, consanguinity, maternal overweight during pregnancy, maternal infection, place delivery, duration of pregnancy, birth order, neonatal jaundice, and sweet and vegetable intake, excluded from the last step in backward stepwise regression model.
